# Examining the Relationship of Online Social Networking Sites’ Activities, Customers’ Brand Choice, and Brand Perception in Health-Related Businesses

**DOI:** 10.3389/fpsyg.2020.546087

**Published:** 2020-10-23

**Authors:** Mehrab Nazir, Jian Tian, Iftikhar Hussain, Adeel Arshad, Muhammad Afzal Shad

**Affiliations:** ^1^School of Economics and Management, Jiangsu University of Science and Technology, Zhenjiang, China; ^2^Faculty of Computing & Engineering, University of Kotli Azad Jammu and Kashmir, Azad Jammu and Kashmir, Pakistan; ^3^Department of Business Administration, University of Kotli Azad Jammu and Kashmir, Azad Jammu and Kashmir, Pakistan; ^4^Department of Commerce, University of Kotli Azad Jammu and Kashmir, Azad Jammu and Kashmir, Pakistan

**Keywords:** health, customer perception, brand choice, brand perception, social networking site

## Abstract

The purpose of this study was to examine the effect of online social networking site activities on brand choice for health-related businesses; the study also explored the mediating impact of brand perception on the connection of online social networking site activities and brand choice in health-related businesses. A self-administered questionnaire was used for data collection from 300 customers, randomly selected from health sector businesses in Pakistan. The findings indicate that online social networking site activities had a substantial influence on customers’ brand choice in health-related businesses and that brand perception mediates the connection between online social networking site activities and customers’ brand choice in health-related businesses. The research also recognized the increasing significance of online social networking sites in health-related businesses. The study offers insights for health-related companies and their managers on visualizing brand perception as well.

## Introduction

Today, health businesses are very conscious in understanding the components that impact the improvement of brand value as well as contribute in creating brand decisions (Loss et al., 2014; Raji et al., 2019). The basic objective of any firm is to construct a healthy and profitable relationship with customers and gain a competitive edge in the market (Bapat and Thanigan, 2016). Businesses have adopted various marketing strategies to retain customers and overcome the level of customers moving from a particular brand to a substitute brand (Zhang et al., 2014). In developing countries like Pakistan, customers shift their choices quickly from one brand to another. Consequently, researchers have been concentrating on brand loyalty and its relationship with various business areas, for example, purchase intention, purchase behavior, and marketing strategies (Abbasi et al., 2011).

The advancement in social media usage has modified communications and has significantly affected marketing strategies. The emerging uses of social media activities like Facebook, Instagram, YouTube, and others in customers’ lives have increased digital posting. Consequently, customers have engaged themselves with social media, and it has increased the percentage of users publishing public messages. This implies that brand-related collaborations and experience to marketing efforts progressively occur inside social media sites (Schivinski and Dabrowski, 2015; Mandhyan and Kumar, 2020).

There are several social networking sites and their applications that allow users to make their profile and share contents with others. Some social media sites offer personal profile creation and allow users to share their views, photographs, and other activities. In contrast, Twitter is a social site that lets the community share short communications. Currently, social networking sites have become an essential communication channel (Evans et al., 2014; Garg and Pahuja, 2020). Circulation of online activities shifted into an innovative type of social communication and has become a need. Traditional shopping has turned into online shopping, and social networking sites have designed a new forum on behalf of customers for online shopping. Customers are now purchasing products online according to their needs.

The most used social networking sites are Facebook, Twitter, and YouTube around the world; that is why marketers are trying to focus fully on these social networking sites to promote their company products cost-efficiently. These social networking sites are not just becoming a source of communication between the customers and these companies but are also creating a better relationship between the firm and their customers (Harridge-March and Quinton, 2009; Kujur and Singh, 2020). Marketers use mostly Facebook as a social media site to represent their ads to influence users to buy their products. Dealers have still perceived the need for manufactured goods, personalization, and targeting of consumers through the internet (Crosby and Johnson, 2000). According to Brodie et al. (2013) and Sharma et al. (2020), social media helped companies in engaging and recognizing their customers and helped them to connect with loyal customers and motivate them. The meaning of a brand is an exclusive tag, period, arguments, symbol, strategy, a mixture of these, or any other representative that identifies the products and services of a company and distinguishes them from those of the competitors (Rosman and Stuhura, 2013; Borah and Tellis, 2016).

With the passage of time, new approaches and patterns of advanced technologies started to influence conventional ways of doing trade. If organizations and traditional businessmen are unaware of the latest technologies and the effects of these digital technologies on businesses, such businesses may fail. Nokia was one of the most common and reliable brands of the phone industry in the 1990s (Bouwman et al., 2014). Due to its reluctance in adopting innovative technologies, Nokia is no longer a rival in the mobile industry. Similarly, the conventional businessman is facing several challenges today as majority of young buyers have been leaning toward virtual shopping (Krbová and Pavelek, 2015; Shroff, 2020).

Pakistan, being an emerging economy, is also facing the same sort of issues. Therefore, this study was designed to identify the effect of social media activities on consumer brand choice in Pakistan and to identify the influences of social media activities on brand perception on three levels (brand awareness, brand consciousness, and brand identification) and also to examine the mediating role of brand awareness, brand identification, and brand consciousness toward the brand choice created by social media activities.

## Literature Review

### Social Networking Site Activities

Social networking sites are defined as the “result of internet-based applications that construct an online relationship, joint work, or sharing contents on the advanced foundation of web 2.0” (Berthon et al., 2012; Jena et al., 2020). More clearly, social networking sites are platforms used to assemble online networks where clients can easily access and associate with one another (Aydin, 2020; Li et al., 2020). Social media consists of online channels that have become a source of communication and participate in an assortment of different activities. It has also become an undeniable significant path for brands to communicate with an appealing audience of the company (Murdough, 2009; Kumar and Singh, 2020). Recently, marketers have grasped social media for an assortment of different marketing objectives like exploring new clients, branding products, promoting sales, and making a strong customer relationship. As indicated by the 2013 social media industry report (Stelzner, 2013), approximately 85% of marketers acknowledged that social networking sites had substantially become mechanized promoters.

Social media provides a platform for brand owners to use behavior and psychographic data from the client’s profile to investigate the impact of the publicity campaign. Conversely, for some years, companies started to understand the loyal audience through social media sites that have engaged in appropriate online networking activities, for example, sharing information and constructing different activities (Song et al., 2019; Singh, 2020).

Techniques for marketing communication have been compelled to change with the advent of the internet and online networking sites, for instance, Facebook, YouTube, and Twitter (Dev et al., 2010; Line et al., 2020). Social media sites have become an incredible source of “word of mouth” statements. Social media consists of different social networking sites in which clients share their understandings and awareness with others. If the customers are satisfied with brands, then it can impact positively on brand choice; conversely, if there are customers unsatisfied with the brands, there can be a negative impact on company loyalty (Trusov et al., 2009; Ahmed et al., 2020). Moreover, social media sites influence the consumers’ buying decision process.

The successful brands have changed their communication strategies and adopted social media sites to attract new customers and clients. Social media sites are becoming a value source in attracting customers and bringing revenues to successful brands. Advertising companies are utilizing social networking sites, for example, Facebook, YouTube, and Twitter, to evaluate their brands’ choices. Numerous fashion houses make their own Facebook and Twitter accounts to communicate with clients without any limitation to increase customer brand choice. Along with these strategies, customers and brands would work together to make new products and service plans for achievement and quality. Brands can build exposure and a strong relationship with clients to increase brand choice (Kim and Johnson, 2016).

Social networking sites play a significant role via online activities in building up personal relationship with clients and providing organizations with opportunities to track clients (Kelly et al., 2010). The rapidly increasing use of social networking sites in our life has caused researchers to examine the utilization of social media to get the fundamental logic behind people’s use of social networking sites, their session duration on these sites, and the number of users using these online sites (Aksoy et al., 2013; Rosen et al., 2013). Kim and Ko (2012) studied the various characteristics of online social media activities of customers, which were summarized into specific attributes such as social connectivity with the clients, just-in-time information about the running products and then luxury brands, perceived value, and trust. Sano (2015) conducted her research on these activities and examined the insurance sector; however, this study will focus on the health-related brands in Pakistan.

Social collaboration means online connectivity with customers according to the aspects of social responsibility on social media through different networking sites. Social connectivity attached customers and businesses to increased brand choice (Lee et al., 2017). According to the classification of Bryce and Fraser (2013), different companies receive benefits when using social networking site activities to share information on the company products and to publicize products, services, and events. It depicts the channels of social media which focus mainly on the relationships and common activities that users participate in with others who share the same interest or identification (Tuten and Solomon, 2014). Social media users fulfill a sense of belonging to the community, identity expression through the community, and a sense of affiliation with others in the community (Dholakia et al., 2009; Casaló et al., 2010).

H1: Social collaboration has a positive effect on brand choice.

### Just-in-Time Information

Just-in-time information refers to the generation, acquisition, and sharing of information by customers. Social networking sites have become an exploration tool to get the most up-to-date information and news about products (Naaman et al., 2011). An objective of this study was to check the trends, as online networking sites have become a source of updated information about brands. At present, consumers mostly follow the running trend, and online networking sites are observed as a new trustworthy source of information (Naaman et al., 2011). Kim and Ko (2012) argued that successful marketing brands are continually updating the latest information about their products on Facebook, Instagram, and YouTube. In this research, trendiness means to distribute the newest and most informative updates about the brands which helps increase consumer brand choice.

H2: Just-in-time information has a positive effect on brand perception

### Perceived Risk

Kim et al. (2008) defined perceived risk as a customer’s belief about uncertainty’s negative consequences on business transactions. Bhatnagar et al. (2000) thought that perceived risk depends on a multidimensional concept such as product risk, financial risk, and information risk. However, social networking sites enable users to set up contact with companies and check new information to overcome uncertainty (Sano, 2014). Perceived risk is a very particular attribute of social media activities, so the researchers selected it as a social media marketing activity component.

H3: Perceived risk has a positive effect on brand perception

### Trust

While trust is a straightforward word, it plays a significant role in online brand choice and purchase intention in the electronic world. In the last 10 years, the general concept of marketing has changed; it has moved from traditional marketing to online marketing. Trust has converted into a significant trademark interconnected with the achievement and dissatisfaction of numerous online businesses (Urban et al., 2009). Trust is an influential factor in relationship congruity between the customer and services. Trust has become a key component of social media activities that can be described as the degree to which the users trust or have faith in the company’s product promotion and that influence customer response toward brand choice. In this way, trust will exist for different kinds of social media activities that provide updated information. Generally, customers choose health-related brand on behalf of other’s using a similar kind of brand or as advised by the physician (Godey et al., 2016).

H4: Trust has a positive effect on brand choice.

### Brand Choice

Brand choice is an essential consequence of brand evaluations and substantial brand perception. It means that with the existence of several competing brands in the market, consumers tend to prefer and feel more attached to a specific brand based on brand perception. Second, brand perception is a basic element to the selection of any brand for the consumers (Keller and Lehmann, 2006). Brand perception may lead to a greater impact on brand choice in products highly advertised online as compared to those that are low advertised. According to Bhat and Reddy (2001), the brand choice orientation of a brand may be increased by high-quality brand perception through social media activities. Ashley and Tuten (2015) argued that social media activities could be utilized to build brand loyalty and brand awareness, advertise consumer engagement, and stimulate social connectivity with customers about the brand choice. Social media sites have improved the traditional marketing approaches to online marketing. Social media activities are recognizing a link between consumers and marketers, creating new prospects, and are conducted with the objectives of increasing brand awareness (Meraz, 2009).

H5: Brand perception has a positive effect on brand choice.

### Brand Perception

Social media is used as a marketing channel for several advertising activities, including complete and updated information, social connectivity with clients, relationship management, buyer enquiry, and brand promotion. Social networking site activities impact the customer through the entire cognitive procedure starting by structuring brand awareness, improving brand image, and growing brand identification to affect the brand choice. Social media activities increase brand awareness and social connectivity with customers by keeping an actual occurrence to enhance brand choice. Furthermore, social networking site activities are utilized to upgrade brand identification and brand awareness to build up a positive consumer brand choice (Tuten and Solomon, 2014).

Customers select products and brands for their use and for their symbolic status. Brands have profound significance (McCracken, 1989) and serve to assemble customers’ self-identities. Buyers use branded products to build their identities, present themselves to other people, or accomplish their identity objectives (Escalas and Bettman, 2003). Many companies are using social networking site activities to increase brand identification for enhancing customers’ brand choice. Social media marketing gives information about brand identifications and their influences on brand choice. More exactly, social media activities play a significant role in determining the effects of brand identification on brand choice.

Social media activities increase the elevated level of customer brand choice by promoting the belief that the brand is very high in symbolic status and reputation; therefore, customers prefer purchasing popular and expensive brand products. Additionally, social media activities give buyers a feeling of recognition with items and diminish risk in buying (Lehmann and Winer, 1997). Earlier studies exhibited that conventional publicizing tools (newspaper, radio, and television) beneficially affect the numerous structures of a brand characteristic such as brand choice, brand loyalty, and brand awareness. Nowadays, buyers have turned into social media and utilize the internet increasingly to stay updated; also, they consider it more reliable than conventional marketing (Mangold and Faulds, 2009). Brand perception has a theoretical interrelationship between the dependent variable (DV) (brand choice) and independent variables (IDVs) (social collaboration, just-in-time information, interaction, and trust) in this research; according to available literature, there is no direct relationship of brand perception with DV and IDV, but brand perception creates a significant mediating effect between DV and IDV.

H6: Brand perception mediates the effect of social collaboration and brand choice.

H7: Brand perception mediates the effect of just-in-time information and brand choice.

H8: Brand perception mediates the effect of trust and brand choice.

H9: Brand perception mediates the effect of perceived risk and brand choice.

## Materials and Methods

### Theoretical Framework

**FIGURE 1:**
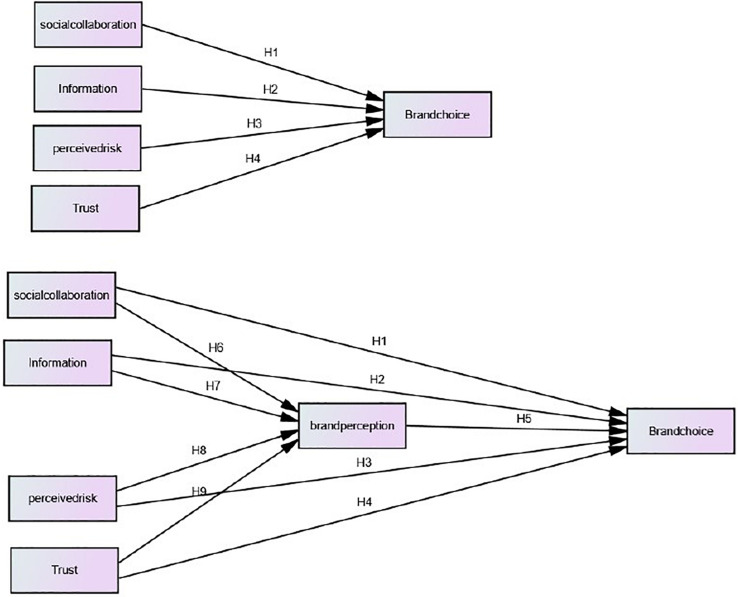
Direct and indirect relationships.

The main characteristics of social networking site activities are production and the consumption of desired content without the constraints of time or location. This research indicates that social media, through the active and aggressive participation of consumers, has a more significant impact on the way consumers behave or think regarding brands than one-sided communication led by a company. Kim and Ko (2012) studied the various characteristics of online social media activities of consumers, which were summarized into specific attributes such as social connectivity with the clients, just-in-time information about the running products and then luxury brands, perceived value, and trust, which increase the efficiency of brand perception and brand choice. Figure 1 illustrates the overall model or framework of the current research and identifies all the relationships between IVs, DV, and MVs.

### Sample and Data Collection

This section discusses the methodology, including sample selection, data description, data collection, regression model, and data analysis. This research focused on health-related products in Punjab, Pakistan. The data were collected through an online survey panel in Punjab, Pakistan. Health-related products have been selected because they are common and because people mostly use online network platforms to acquire them in Pakistan.

Moreover, in the case of the health industry, consumers spend enough time in the selection of the brand. The research was conducted on social networking sites, so a non-probability sampling technique was used. The non-probability sampling was selected as the researchers took the sample from an open market. Then the final questionnaire was distributed to a sample of 300 respondents for data collection. Nunnally (1994) endorsed a minimum sample size of 300 for a sufficiently accurate evaluation of the Cα coefficient. Several studies have used and recommended a sample size of 300 for applying structural equation modeling (SEM) analysis; therefore, the researchers in this study have taken a sample size of 300 (Krejcie and Morgan, 1970; Comrey and Lee, 2013; Lien et al., 2015; Suki, 2016). The sample was obtained through a non-random sampling technique and analyzed with various statistics using SPSS and SEM. The researchers used a Likert scale of 5 points. The hypotheses were measured based upon a scale by Ko et al. (2005).

Table 1 indicates that gender is deliberated as an imperative demographic variable in the research of online advertisement because the companies manufacture products for both females and males according to their demand. Therefore, online advertisements are made differently for both females and males by businesses. The results of the study showed that 84.7% of respondents were male and 15.3% were female. Age plays a significant character in the decision-making practice, so age was considered as a demographic variable. It revealed the understanding level of persons. Table 1 indicates that 35.3% of the total respondents were less than 25 years, 34.3% were between the ages of 25 to 45 years, and 30.3% were 46 years and older.

**TABLE 1:** Demographic analysis (percentage).

Variables	Category	City		Overall
		
		Faisalabad	Lahore	
Gender	Male	94.7	74.7	84.7
	Female	5.3	25.3	15.3
Age (years)	Younger than 25	29.3	41.3	35.3
	25–45	27.3	41.3	34.3
	46 year and older	43.3	17.3	30.3
Marital status	Single	47.3	62.0	54.7
	Married	52.7	38.0	45.3
Family size	1–2	26.7	16.0	42.7
	3–5	44.0	45.3	44.7
	6–10	16.7	28.7	22.7
	More than 10	12.7	10.0	11.3
Education	Matriculation	15.3	4.7	10.0
	Intermediate	16.7	9.3	13.0
	Bachelor’s	40.7	51.3	46.0
	Master’s	22.0	30.0	26.0
	Ph.D.	5.3	4.7	5.0

Marital status is also an important demographic characteristic on which basis marketers make online advertisements through different ways to attract their customers. The finding of the study shown in Table 1 illustrates that 54.7% of the entire respondents were single, while 45.3% were married. The results of the study showed that 49% of the total respondents completed their bachelor’s level, 46% obtained a master’s degree, 18% had post-graduate degree, and 32% had diplomas in their field of interest.

## Results and Discussion

Table 2 indicates that the estimation model was created to calculate confirmatory factor (CF) analysis and the maximum likelihood method conducted for model estimation. As we utilized a setup estimation scale, CF analysis was calculated in this research, and reliability exploration enables one to contemplate the possessions of estimation scales. The reliability examination technique ascertains several items, usually used for the estimation of measure. The alpha (Cronbach) model was utilized, which expresses the within-item consistency because of the average within-item relationship. Reliability depicts how well the 24 constructs of composite reliability are estimated, and the value of each item ranges between 0.75 and 0.91, which illustrates a good indication of internal consistency. CFI of between 0.87 and 1.00 shows good model fit; consequently, it specifies the validation of unidimensional model (Sureshchandar et al., 2002). By assessing the factor loadings, the convergent validity of the each estimation stuff was evaluated, and composite reliabilities as standard factor loadings of all measures are between 0.51 and 0.86, which exceed the standard value of 0.5 (Hair et al., 1995, 2010). According to the statistical estimation result of Table 2, the composite reliability of every variable varies from 0.699 to 0.891, which shows that all indicators are approximately under the threshold level. Additionally, the values of average variance extracted of each construct ranged from 0.51 to 0.71, which indicates that all the values are under the threshold level (Fornell and Larcker, 1981); these results indicate the worth of the instrument used for advanced analysis.

**TABLE 2:** Results of confirmatory factor analysis (CFA) model.

	Factor loading	Reliability	CR	AVE	CFI
SC1	0.63	0.75	0.745	0.54	0.97
SC2	0.64				
SC3	0.63				
SC4	0.50				
JIT1	0.65	0.80	0.789	0.59	1.00
JIT2	0.52				
JIT3	0.75				
JIT4	0.71				
PR1	0.66	0.70	0.691	0.51	1.00
PR2	0.69				
PR3	0.65				
PR4	0.59				
TR1	0.65	0.75	0.744	0.55	0.90
TR2	0.60				
TR3	0.62				
TR4	0.76				
BP1	0.70	0.84	0.829	0.65	0.87
BP2	0.55				
BP3	0.58				
BP4	0.75				
BC1	0.86	0.91	0.891	0.71	0.95
BC2	0.51				
BC3	0.82				
BC4	0.75				

### Structure Equation Modeling

Table 3 presents the connection between all the endogenous and exogenous variables of this research. This empirical and analytical study, using SEM, investigated the effects of social networking sites on brand choice, as mediated by brand perception. Table 3 illustrates the main results of SEM analysis.

**TABLE 3:** SEM analysis.

			Estimate	*SE*	C.R.	*p*	Decision
Brand perception	←	Social collaboration	0.201	0.056	3.729	***	Supported
Brand perception	←	Information	0.205	0.051	3.804	***	Supported
Brand perception	←	Perceived risk	0.187	0.046	3.458	***	Supported
Brand perception	←	Trust	0.223	0.035	4.227	***	Supported
Brand choice	←	Brand perception	0.214	0.043	3.866	***	Supported
Brand choice	←	Trust	0.132	0.043	2.541	0.011	Not Supported
Brand choice	←	Social collaboration	0.155	0.043	2.938	0.003	Supported
Brand choice	←	Information	0.126	0.039	2.376	0.018	Not Supported
Brand choice	←	Perceived risk	0.110	0.058	2.041	0.041	Not Supported

***Means p value is significant at 0.05 level.

The research data investigating health-related product users were analyzed using the SPSS 20 and the AMOS 24 statistics package programs to examine the hypotheses in this research. SEM was used to examine the results of the hypotheses. The outcomes were inside the acceptable ranges (chi-square = 329.471, *df* = 193, CMIN/DF = 1.707, *p* < 0.05, AGFI = 0.844, GFI = 0.911, RMR = 0.040, CFI = 0.853, TLI = 0.824, and RMSEA = 0.049), associating the model’s goodness of fit used in the research. According to goodness-of-fit model, social networking site activities affected brand choice with mediation of brand perception in the health industry.

According to the H1 analysis, it is estimated that β = 0.201, C.R. = 3.729, *p* < 0.05; hence, researchers accepted the hypothesis, which means that social collaboration has significantly affected brand perception toward brand choice. The results showed that online connectivity with users increased brand perception of increasing brand choice. It implies that social networking sites have broad impacts for marketers to make their quality attachment with customers to increase brand perception for their products, bringing about expanded sales. In this manner, we accept H1; that is, there is a signification relation between brand preferences toward increased brand choice through social networking site activities.

From the above result of H2, it is assessed that β = 0.205, C.R. = 3.804, *p* < 0.05; thus, we accepted hypothesis H2, which means that social networking sites have become a search tool to convey the most up-to-date information and news about products. It shows that online networking sites have become an immediate connection between customers and the brand. Social networking sites subsequently are a valuable tool to deliver up-to-date information about the different branded products and to create awareness, resulting in expanded utilization of the brand value. So we accept the hypothesis that there is a significant connection between users gathering information about items and brand perception on social networking sites toward brand choice.

Concerning H3, it was estimated that β = 0.187, C.R. = 3.458, *p* < 0.05; thus, we accepted this hypothesis, which means that there exists a customer belief about the consequences for the business transaction. Some researchers said that perceived risk depends on a multidimensional concept such as product risk, financial risk, and information risk. Risk is an activity of the social networking sites according to the views of customers. Customers admitted that social networking sites could overcome the uncertainty and risk of the brand products. The effect of trust on brand perception (β = 0.223, C.R. = 4.227, *p* < 0.05) and the effect of brand perception on brand choice (β = 0.214, C.R. = 3.866, *p* < 0.05) were significant, so we accepted H4 and H5. According to the result of the studied hypotheses, trust can be described as the key factor of social networking site activities which can significantly influence the promotion of customer brand products to influence customer brand perception toward brand choice. With the existence of several competing brands in the market, customers tend to prefer and feel more attached to one of the brands based on brand perception.

**FIGURE 2:**
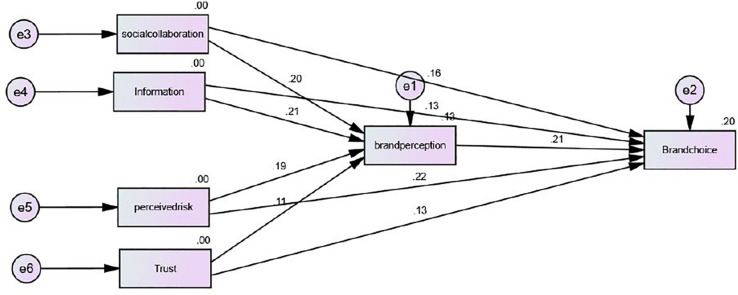
AMOS measurements.

The connections between the developed hypotheses including social collaboration, just-in-time information, perceived risk, and trust of our theory test are shown in Table 4 and Figure 2. However, the values of H6, H7, H8, and H9 illustrated that there exists a strong relationship between social networking site activities

**TABLE 4:** Indirect effects.

	Trust	Perceived risk	Information	Social collaboration	Brand perception
Brand perception	.	.	.	.	.
Brand choice	0.051	0.003	0.002	0.001	.

## Conclusion

This research investigated the effects of health-related social networking site activities on brand choice with the mediating role of brand perception. The contributions of this study based on findings are as follows. Firstly, social collaboration, just-in-time information, perceived risk, and trust have a positive significant effect on brand perception, confirming that social networking site activities contribute to health-related brand perception. Secondly, brand perception has a positive significant effect on brand choice, confirming that health-related products with low brand perception could effectively follow social networking site activities to expand brand choice. In contrast, social collaboration directly increases brand perception and brand choice as well. The findings of this research are compatible with the findings of Aaker (2009) that brand perception had positive significant influences on brand choice, resulting in brand perception playing a mediating role in the relationship between social networking site activities and brand choice. The results also proved that perceived social networking site activities have influenced brand choice. In fact, brands itself allow customers to express their preferences on social networking sites (Wallace et al., 2014). Users who are engaged with majority of the social networking site activities, concerning brand, are more prospectively and warmly attached to the manufacturer, which shows the feeling of loyalty toward brand. Thus, brand marketers should develop different ways to connect and interact with customers through different activities on social networking sites as it would enhance the loyalty of customers to the brand. Social networking site activities are a source to develop a positive impact on brand perception, which subsidizes health-related brand value; the health industry should persuade their customers to utilize social networking sites more effectively by growing more interesting and aggressive social networking site activities.

### Limitations and Implications

This study focuses on some social networking site activities from an entire online networking environment. Due to resource constraints, the researchers could not investigate other online networking site activities, which can provide very good opportunities for the marketers to promote their businesses. The activities of these social networking sites also play a very important role in influencing customers’ purchase intentions and brand choice as well. It is better to accomplish more research on other social networking sites and their applications and compare them and to make sense of the proper marketing channels for various marketers to originate with more extensive marketing strategies for marketers. According to the research, the population is also an important factor in conducting the survey. The research is restricted to exploring all followers of the health industry through social networking sites. The research was conducted in the health-related businesses of Pakistan; there are several other businesses in Pakistan in which social media sites can play an important role in promoting businesses. Therefore, marketers are advised to try to design strategies to work on social media sites and their activities in other sectors. For health-related service and manufacturing companies and policymakers, this research demonstrates that social networking site activities impact the customer through the entire cognitive procedure starting by structuring brand awareness, improving brand image, and growing brand identification to affect brand choice. Health-related companies should post-relevant information on their official pages on Facebook, Twitter, etc. to boost brand perception toward consumer brand choice, because social media activities enrich brand awareness and social connectivity with customers by keeping an actual occurrence to enhance brand choice. Thus, the study’s theoretical model produced a clear estimation and provides a multidimensional model of the statistical relationship among the variables which enhance consumer brand choice in the fast-competitive market.

## Data Availability Statement

The raw data supporting the conclusions of this article will be made available by the authors, without undue reservation, to any qualified researcher.

## Ethics Statement

Ethical review and approval was not required for the study on human participants in accordance with the local legislation and institutional requirements. Written informed consent to participate in this study was provided by the participants through their supervisors, managers and owners.

## Author Contributions

JT and IH: conceptualization and writing—review and editing. MN: methodology, formal analysis, and data curation. AA: software. MN and AA: validation. AS: investigation. IH: writing—original draft preparation. JT: supervision and funding acquisition. All authors contributed to the article and approved the submitted version.

## Conflict of Interest

The authors declare that the research was conducted in the absence of any commercial or financial relationships that could be construed as a potential conflict of interest.
